# The Clinical Course of a Severe Case of Malignant Catatonia

**DOI:** 10.7759/cureus.58142

**Published:** 2024-04-12

**Authors:** Kalashree Gopal, Ryan Liengswangwong, Shyamalee A Ramaraj, Jeremiah Howard, James N Byrd

**Affiliations:** 1 Medicine, Alabama College of Osteopathic Medicine, Dothan, USA; 2 Internal Medicine, Mobile Infirmary Medical Center, Mobile, USA

**Keywords:** electroconvulsive therapy, nmda antagonists, benzodiazepines, dysautonomia, malignant catatonia

## Abstract

Catatonia is a psychomotor syndrome predominantly associated with mental illness disorders, most commonly bipolar disorder and schizophrenia. Catatonia is classified as malignant when, in addition to catatonic symptoms, dysautonomia is present. Autonomic abnormalities can include changes in temperature, labile blood pressure, and changes in heart and respiratory rates. Because malignant catatonia is life-threatening, prompt recognition and management are essential to prevent mortality. We present a severe case of catatonia with malignant features that highlight the importance of early diagnosis and treatment.

## Introduction

Catatonia is a collection of symptoms associated with several mental illness disorders, the most common being bipolar disorder and schizophrenia. The pathophysiology of catatonia is still unclear, although studies suggest it may be a result of dysfunction of motor circuits [1], reduced gamma-aminobutyric acid (GABA) activity in the right lateral orbitofrontal and right posterior parietal cortex [2], glutamate hyperactivity in the basal ganglia [3], as well as a strong dopamine D2 receptor blockade [4]. Symptoms of catatonia listed in the Diagnostic and Statistical Manual of Mental Disorders, Fifth Edition (DSM-5) include stupor, waxy flexibility, catalepsy, mutism, posturing, negativism, stereotypes, mannerisms, grimacing, agitation, echopraxia, and echolalia. Three or more of these symptoms are required for diagnosis [5], and a diagnosis of malignant catatonia is made when these symptoms are accompanied by fever and dysautonomic symptoms such as hyperthermia, tachycardia, and fluctuations in the blood pressure [6]. Lorazepam is the first-line treatment for catatonia and may be administered from a single dose to as long as symptoms continue [7]. For malignant catatonia, a single-dose lorazepam trial may be instituted. If the patient remains catatonic, electroconvulsive therapy (ECT) is the next best step in treatment and is often lifesaving [4,6,7]. This case report discusses the theorized pathophysiology behind catatonia, symptomatic recognition, as well as diagnosis and management. Given that the mortality rate of malignant catatonia is 9%-20% [6], we hope to emphasize and raise awareness of the importance of early recognition and treatment.

## Case presentation

A 32-year-old homeless man presented to the emergency department (ED) via an ambulance. Emergency Medical Services (EMS) reported that they found him lying in a field, smelling as though he was covered in urine and feces, and refusing any verbal interaction. Upon presentation to the ED, the patient appeared awake but would not speak to anyone, make eye contact, answer any questions, or follow any commands. He nodded occasionally and did respond nonverbally to some questions, mainly regarding food needs. He would spike low-grade fevers, around 101°F-102°F, and there was some rigidity and posturing present. The patient maintained his right arm in an upright position and was unable to relax.

On day three of his hospitalization, he was given lorazepam (2 mg intravenous (IV)) to evaluate his rigidity and mutism and rule out catatonia. He responded well and was soon sitting up, eating, and conversing normally. He reported that he had been diagnosed with bipolar disorder and schizophrenia in the past. He was placed on lorazepam (2 mg oral three times daily) as well as olanzapine (10 mg at bedtime). At this point, while he continued to have intermittent hyperthermia and tachycardia, his blood pressure remained stable. Lumbar puncture ruled out autoimmune encephalitis, and head computed tomography (CT), brain magnetic resonance imaging (MRI), and electroencephalogram (EEG) were unremarkable, raising more concern for malignant catatonia.

Over the next few days, his altered mentation persisted, and it became clear that he was not responding appropriately to the oral lorazepam. This was likely secondary to his lack of oral intake. Since antipsychotic medications can exacerbate negative symptoms, the olanzapine was discontinued, and he was given another dose of IV lorazepam. This temporarily improved his mental status, and he was later transferred to the intensive care unit (ICU) to consistently receive IV lorazepam. ICU nurses reported that the effects lasted approximately 30-60 minutes, which was less than expected. There was still some concern for intermittently spiking temperatures, tachycardia, and hypertension, and the diagnosis of malignant catatonia was made. At this point, ECT was concluded to be the best course of action, although an accepting facility that performed ECT could not be located.

Since the patient continued to deteriorate on a total of 6 mg of lorazepam daily, his dose was increased to 8 mg (2 mg IV four times daily); he was also started on memantine (10 mg). Since there was a lack of improvement on 8 mg of lorazepam daily, his dose was increased to 3 mg four times daily or 12 mg/day total. This occurred on day 15 of his hospitalization. Over the next few days, notable improvement was evident. His oral intake improved, and he showed increased alertness and intermittently engaged in conversation. As a result, and because 12 mg of lorazepam per day is a significant dose, it was reduced to 8 mg daily, and then to 6 mg daily. Divalproex (500 mg twice daily) was also initiated for his underlying bipolar disorder. He was consistently monitored for any decompensation, and his mental status improved daily, despite the dose decrease. Ultimately, since the patient required continued management with benzodiazepines, he was transferred to an inpatient psychiatric unit on day 45 of his hospital stay.

## Discussion

According to the DSM-5, three or more of the following symptoms are required for a diagnosis of catatonia: stupor, waxy flexibility, catalepsy, mutism, posturing, negativism, stereotypes, mannerisms, grimacing, agitation, echopraxia, and echolalia [5]. In our patient, mutism or inability to speak was evident upon presentation. He additionally presented with waxy flexibility and posturing, holding his arm up for an extended length of time, as well as catalepsy, which refers to a patient’s limbs remaining in a fixed, unnatural position. Negativism, or a lack of response to external stimuli, was also present. Since four criteria were met, the patient was diagnosed with catatonia. He was diagnosed with malignant catatonia due to autonomic anomalies, specifically his labile blood pressure readings, spiking temperatures, and tachycardia.

Another useful tool in diagnosing catatonia is the lorazepam challenge test. This is performed by administering one or two 1-2 mg IV boluses of lorazepam. The patient is typically assessed after 15 minutes, and if there is a reduction in catatonic symptoms by over 50%, the test is considered positive. A positive test can be used to confirm a diagnosis of catatonia; however, catatonia cannot be ruled out if the patient does not respond [8]. Because our patient did present with some signs of catatonia, he was given 2 mg of lorazepam by IV. He responded with a marked reduction in symptoms and was able to provide additional psychiatric history. While catatonia was suspected due to his clinical symptomology and presentation, the lorazepam challenge test proved very useful because we were able to confirm his diagnosis and proceed with benzodiazepine treatment which was ultimately helpful to the patient. 

In addition, our patient conveyed his desire to be on his prior dose of 10 mg of olanzapine at bedtime. However, once catatonia is diagnosed, antipsychotics should be discontinued as they can cause or worsen catatonia [6]. Additionally, neuroleptic malignant syndrome, a pharmacologically induced form of malignant catatonia, can occur with exposure to dopamine antagonist antipsychotic drugs. Evidence also suggests that a D2 dopamine receptor blockade is directly related to the risk of exaggerating catatonia and giving rise to malignant features [4,8]. Since our patient was highly unlikely to be taking neuroleptics prior to his presentation, we did not suspect neuroleptic malignant syndrome. However, due to his lack of improvement with lorazepam, his olanzapine was discontinued to remove the possibility of it intensifying his catatonia.

Lorazepam is the most widely administered medication for catatonia. Reduced gamma-aminobutyric acid type A (GABA-A) receptor activity in the right orbitofrontal and posterior parietal cortices is postulated to be one of the causes of dysfunction. Because GABA is an inhibitory neurotransmitter, this likely leads to overactivity of these two cortices, resulting in the motor and affective symptoms seen in catatonia. Since benzodiazepines stimulate GABA-A binding and therefore lead to increased inhibitory signaling, this would lead to decreased neural activity and improved symptoms [2]. However, it bears repeating that this mechanism has never been explicitly studied. Lorazepam doses of 2-16 mg/day have been reported without significant sedation, and effects typically wear off after 3-5 hours and subsequently return [7]. Our patient was initially started on 6 mg of lorazepam per day, and his dose was eventually titrated up to 12 mg per day, which is quite a significant dose. In addition, its effects were short-lived, lasting only 30 minutes to one hour. The reasons for this are unclear; however, our patient would eat and drink during this brief lucid interval, and it is likely that this is what prevented him from decompensating medically.

Our patient was also started on memantine, while he was continuing to deteriorate on 6 mg of lorazepam per day. N-methyl-D-aspartate (NMDA) antagonists memantine and amantadine may be considered for catatonia that is refractory to benzodiazepines and when ECT is unavailable. Doses of 5-20 mg of memantine daily have been reported in literature and have been used in conjunction with benzodiazepines as well as independently [8]. When patients unresponsive to benzodiazepines were successfully treated with NMDA antagonists, recovery occurred relatively gradually. Theoretically, amantadine and memantine may modulate glutamate-mediated cortical and subcortical connectivity. However, the gradual response to these agents suggests that NMDA receptors are only secondarily involved in catatonia while GABA-A receptors are primarily involved [3]. While multiple case reports have discussed instances in which NMDA antagonists have proven clinically beneficial [9,10], we are unaware of any studies that confirm the effectiveness of NMDA antagonists. This is an area that would benefit from further research.

## Conclusions

According to the DSM-5, catatonia is a collection of symptoms including at least three of the following: stupor, waxy flexibility, catalepsy, mutism, posturing, negativism, stereotypes, mannerisms, grimacing, agitation, echopraxia, and echolalia. Malignant catatonia occurs when these symptoms are accompanied by dysautonomia. As a result of its autonomic dysfunction, malignant catatonia is potentially fatal, and early recognition and treatment are of the utmost importance. Our patient presented with mutism, waxy flexibility, catalepsy, as well as negativism. He responded positively to a lorazepam challenge test and was therefore diagnosed with catatonia. As his temperatures would periodically spike and due to his intermittent hypertension and tachycardia, his was concluded to be a malignant presentation. Clinical improvement was ultimately noted once he was placed on a regimen of 10 mg of oral memantine as well as 12 mg of IV lorazepam per day, which was quite a significant dose. In addition to the risk of fatality from autonomic instability, our patient did not eat unless he was given IV lorazepam. This is another reason that treatment likely prevented our patient from declining medically. We hope to emphasize the importance of early recognition and treatment in patients with malignant catatonia, as without treatment, the morbidity and mortality of this condition remain high.
